# Effectiveness of proactive and reactive services at the Swedish National Tobacco Quitline in a randomized trial

**DOI:** 10.1186/1617-9625-12-9

**Published:** 2014-06-03

**Authors:** Eva Nohlert, John Öhrvik, Ásgeir R Helgason

**Affiliations:** 1 Centre for Clinical Research, Uppsala University, Västmanland County Hospital, Västerås 721 89, Sweden; 2 Department of Medicine, Karolinska Institutet, Stockholm, Sweden; 3 Department of Public Health Sciences, Social Medicine, Karolinska Institutet and Centre for Epidemiology and Community Medicine, Stockholm County Council, Stockholm, Sweden; 4 Reykjavik University, Reykjavik, Iceland

**Keywords:** Tobacco cessation, Smoking cessation, Quitline, Telephone counselling, Intensity

## Abstract

**Background:**

The Swedish National Tobacco Quitline (SNTQ), which has both a proactive and a reactive service, has successfully provided tobacco cessation support since 1998. As there is a demand for an increase in national cessation support, and because the quitline works under funding constraints, it is crucial to identify the most clinically effective and cost-effective service. A randomized controlled trial was performed to compare the effectiveness of the high-intensity proactive service with the low-intensity reactive service at the SNTQ.

**Methods:**

Those who called the SNTQ for smoking or tobacco cessation from February 2009 to September 2010 were randomized to proactive service (even dates) and reactive service (odd dates). Data were collected through postal questionnaires at baseline and after 12 months. Those who replied to the baseline questionnaire constituted the study base. Outcome measures were self-reported point prevalence and 6-month continuous abstinence at the 12-month follow-up. Intention-to-treat (ITT) and responder-only analyses were performed.

**Results:**

The study base consisted of 586 persons, and 59% completed the 12-month follow-up. Neither ITT- nor responder-only analyses showed any differences in outcome between proactive and reactive service. Point prevalence was 27% and continuous abstinence was 21% in analyses treating non-responders as smokers, and 47% and 35%, respectively, in responder-only analyses.

**Conclusion:**

Reactive service may be used as the standard procedure to optimize resource utilization at the SNTQ. However, further research is needed to assess effectiveness in different subgroups of clients.

**Trial registration:**

ClinicalTrials.gov: NCT02085616

## Background

Tobacco control remains a critical public health challenge, and encouraging smoking cessation is crucial to reducing mortality and morbidity [1]. Evidence-based smoking cessation treatments exist but are underutilized, and an important issue is to meet the individual needs of the smokers [2]. A number of problems and barriers for cessation work and methods are identified among health-care professionals as well as among smokers [3,4]. Telephone counselling is an evidence-based option that is both effective and cost-effective [2,5-9]. Quitlines have an extensive range, the potential to reach underserved populations, and an important function in supporting the health-care system to help patients quit smoking [2,6]. They are usually free of charge for the caller and offer both a reactive service, in which only incoming calls are attended, and a proactive service, which offers a number of callbacks [5]. According to meta-analyses, the odds of quitting are 40% higher for smokers who call quitlines and receive multiple proactive counselling than for controls who receive brief counselling or mailed self-help materials. There is evidence of a dose–response relationship [2,10] and clearer evidence of benefits for smokers who are motivated to quit, but no difference is found between different types of counselling methods and adjunctive self-help materials [2,5,10]. However, in the most recent update there are limited evidence about the optimal number of calls [9], and for the English national quitline, the additional effect of offering more intensive proactive counselling compared with standard care is unclear [11,12]. Because there are considerable differences in quitline treatment protocols, organization, and techniques in different countries, yet, international comparisons are difficult [8,10,13].

In Sweden, the prevalence of adult daily smoking has steadily declined since the 1980s to 11% in 2012. Yet, 1.6 million Swedes use tobacco (cigarettes and/or snus [moist snuff]) every day, and 6,600 persons die in smoking-related diseases every year (18 per day) [14]. The supply of smoking cessation support will be the most important component of the tobacco control work to reduce smoking-related mortality in coming decades. However, the need for cessation support is greater than can be managed with the present resources. Furthermore, there are large regional differences in the supply of cessation support [15].

The Swedish National Tobacco Quitline (SNTQ) is a nationwide, free service operated by the Stockholm County Council Health Service and financed by the Swedish Government. The quitline started in 1998 with a reactive service. In 1999, a proactive service was introduced, and clients could choose a reactive or proactive service. Previous studies report about a 30% point prevalence abstinence (responder-only) at 12-month follow-up, a cost per life-year saved of about 400 USD, and that the proactive service is significantly more effective than the reactive service for women but not for men [16,17]. However, these results are based on non-randomized studies in which clients could choose which service they wanted.

Given the reach of the quitline to meet the increased national demand for cessation support, and its current funding constraints, it is crucial that the most clinically effective and cost-effective service is identified. To study the effectiveness of the two services at the SNTQ, a randomized controlled trial (RCT) started in 2009. The aim was to compare the effectiveness of the high-intensity proactive service with that of the low-intensity reactive service.

## Methods

### Standard SNTQ process

The SNTQ operates two or three lines 51 hours per week, Monday-Thursday 9 am -8 pm and Friday 9 am-4 pm. The number of calls per year is about 10,000 but fluctuates quite a lot with varying marketing activities. About 40% are not treatment calls; these include e.g., brief questions, and calls from relatives and health-care personnel. The number of new treatment clients is normally about 2000 per year. All calls are registered in a computerized database. When a tobacco user calls to discuss his/her own tobacco behaviour, the counsellor asks whether the client would like to sign up for cessation support. If the client gives verbal consent, their preference of callback (proactive service) or no callback (reactive service) is recorded, and a registration form, which includes the baseline questionnaire, is mailed to them. A returned baseline questionnaire is regarded as informed consent and the client is included in a study base to assess effectiveness. The baseline information is registered in computerized client records in accordance with common rules of confidentiality. Printed material partly tailored to the client’s needs and motivation to quit is offered free of charge. Twelve months after the first call, a follow-up questionnaire is sent by mail to the client. Non-responders to the baseline or follow-up questionnaire receive up to two reminders, one by mail, and one by telephone.

### Counselling

The counsellors are trained health professionals, such as nurses, dentists, dental hygienists, or psychologists, with previous experience of primary and secondary prevention. Additionally, all counsellors receive approximately 6 months of training in tobacco cessation methods. The structured treatment protocol is a mixture of motivational interviewing (MI), cognitive behaviour therapy, and pharmacological consultation. Regular call monitoring with supervision is performed for quality assurance and an independent university-based coding laboratory also assess the quality (fidelity) of MI. Twenty counsellors offered treatment during the study period, of which eight had more than 20 clients, nine had 5–20 clients, and three had less than five clients.

### The present RCT

**Figure 1:**
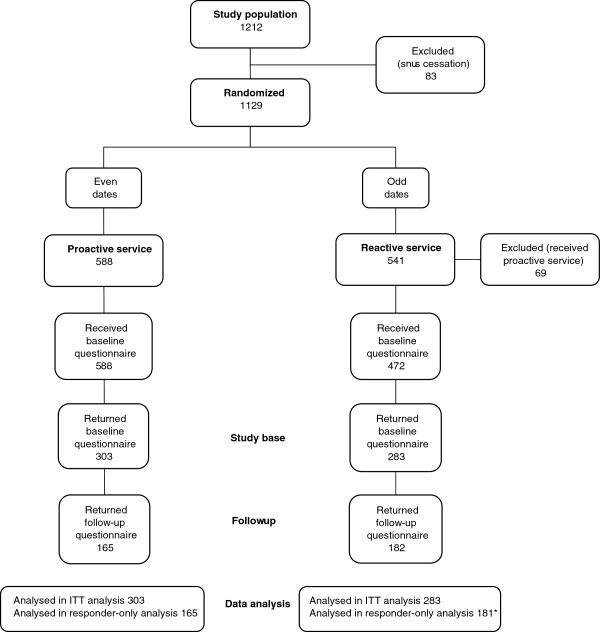
**Flow chart of the study.** Clients included from February 2009 to September 2010, inclusive. *Internal drop-out for outcome variables in the follow-up questionnaire for one individual.

In total, 1212 calls to the SNTQ were classified as new treatment calls during the inclusion period from February 2009 to September 2010, inclusive. Calls from clients who had called some time during the past ten years were not classified as new treatment calls. Snus cessation calls were excluded, leaving 1129 for randomization, 588 to proactive service and 541 to reactive service. The study was performed within the normal run of the SNTQ with the only difference that callers were not offered a choice of callbacks or no callbacks. Instead were only those calling on even dates offered callbacks, thus proactive service, and those calling on odd dates were informed that they could themselves call back whenever they liked, thus reactive service. The only difference between the two treatment groups was the offer to be called back. As the randomization was performed at the time for the clients’ first call, the intervention has started and was known by the clients when they decided to return the baseline questionnaire and thus be included in the study base, giving the study a semi-randomized character. Of callers randomized to proactive service, 73% wanted to be called back. Of callers randomized to reactive service, 13% received callbacks. As the proactive service implies an offer to be called back, both those who did and those who did not want to be called back were included in the study. However, the reactive service implies that there is no offer to be called back, so those who received callbacks were excluded from the study. The study base consisted of the 586 clients who returned the baseline questionnaire, 303 of whom were allocated to the proactive service group and 283 to the reactive service group (Figure 1).

### Questionnaires and outcomes

The questionnaires included questions about the use of cigarettes, snus, and pharmaceuticals, different aspects of present and previous smoking habits and quitting attempts, exposure to second-hand smoke, and access to other support (social, professional). The baseline questionnaire also included three questions that asked the participants to rate themselves on a Visual Analog Scale from 1 = “not at all likely” to 10 = “very likely”. The questions were 1) “The probability that I will be completely smoke-free in one year?” 2) “I can handle stress and depression without smoking” and 3) “I will use pharmaceuticals to control tobacco cravings if necessary”. Three further questions in the baseline questionnaire assessed to what extent the client experienced that the counsellor 1) was a keen listener, 2) tried to understand the client’s needs, 3) showed respect for the client’s own goals. The response alternatives were “a lot”, “quite a lot”, “to some extent”, and “not at all”. “A lot” for all the three questions was required for “high” level of satisfaction in the analyses.

Abstinence was assessed with two questions: 1) “Have you smoked (one or more deep drags) during the past 7 days?” with response alternatives of “no, not at all”, “yes, but not daily”, and “yes daily”; and 2) “When did you take your last puff?” with response alternatives of “0-7 days ago”, “more than 7 days but less than 6 months ago”, “6-12 months ago”, and “more than 12 months ago”. Outcome measures were point prevalence abstinence (not a puff in the last week) and 6-month continuous abstinence (not a puff in the last 6 months) at the 12-month follow-up.

The study was approved by the Ethical Committee at Karolinska Institutet (Dnr 00–367).

### Data analysis

Power calculations estimated that approximately 2500 smokers were required to detect an expected difference of 5% of units between the arms with 80% power, assuming a point prevalence of 30% in the reactive arm. To compensate for loss to follow-up, it was decided to randomize about 3000 clients. Approximately 19 months into the data collection a preliminary analysis was done, to assess if there were any trends in outcome towards the original hypothesis that would warrant continued data collection in accordance with the previous power calculations. This analysis revealed no effect of the proactive service over the reactive and thus it was decided to discontinue new recruitment to the study.

SPSS (version 20) was used for statistical analyses and statistical significance was set at *p* < .05 (two-sided). Abstinence at follow-up was measured i) according to intention-to-treat (ITT), where non-responders to the follow-up questionnaire were treated as smokers and ii) in responders only. Continuous variables were compared using either parametric t-tests or non-parametric Mann–Whitney U-tests. Categorical variables were compared with chi-square tests. Logistic regression analysis was performed to calculate odds ratios (ORs) with a 95% confidence interval for the two abstinence measures. Univariable analyses were performed for all relevant independent variables, and those found to be statistically significant at *p* < .2 were included in the multivariable analyses with service, gender, and age. The multivariable analyses were performed with forward and backward stepwise selection, to detect potential collinearity that could disturb the analyses, with 5% for inclusion and 5% for exclusion. Hosmer and Lemeshow goodness of fit test was used to test the overall fit of the logistic regression model [18]. Two regression models were performed for each abstinence measure, one predictive that merely included the variables known at baseline and one descriptive that also included variables describing what happened in the subsequent year. From the descriptive models, we had to exclude two variables because of too many missing observations; duration of call number 2 from the point prevalence model, and snus use in the week before 12-month follow-up from the 6-month continuous abstinence model.

## Results

Fifty-nine per cent of the study base returned the 12-month follow-up questionnaire, 55% in the proactive and 64% in the reactive service (*p* = .015). The flow chart of the study is presented in Figure 1.

The typical caller in the present study was a 50-year-old woman with 12 years of education who had been a daily smoker for 30 years. Population characteristics at baseline are presented in Table 1. The number of calls and the total call duration was significantly higher in the proactive than in the reactive service; however, the duration of first call was equal in both services (Table 2).

**Table 1:** Population characteristics at baseline

	**Total**	**Proactive**	**Reactive**	***p*-value**
	**N = 586**	**n**_**pro**_ **= 303**	**n**_**re**_ **= 283**	
**Gender:** women (%, n/N)	78 (457/586)	79 (238/303)	77 (219/283)	.734*
**Age groups** (%, n/N):				
≤ 34	20 (115/576)	21 (64/301)	19 (51/275)	.118*
35-49	25 (142/576)	27 (82/301)	22 (60/275)	
50-64	39 (223/576)	38 (114/301)	40 (109/275)	
≥ 65	17 (96/576)	14 (41/301)	20 (55/275)	
**Education in years** (%, n/N):				
0-9	25 (142/570)	23 (67/296)	27 (75/274)	.400*
10-12	42 (237/570)	42 (125/296)	41 (112/274)	
≥ 13	33 (191/570)	35 (104/296)	32 (87/274)	
**Number of cig/day** (data journal) (%, n/N):				
0	27 (131/482)	24 (60/248)	30 (71/234)	.268*
1-14	34 (163/482)	36 (90/248)	31 (73/234)	
≥ 15	39 (188/482)	39 (98/248)	39 (90/234)	
**No smoking the past seven days at baseline** (%, n/N)	28 (161/583)	26 (78/301)	29 (83/282)	.342*
**Number of years smoked before baseline** (md, q_1−_q_3,_ N)	33, 18–40, 552	30, 16–40, 290	35, 20–43, 262	.024^†^
**Stages-of-change** (%, n/N):				
-precontemplation/contemplation	19 (81/433)	18 (41/229)	20 (40/204)	.872*
-preparation	29 (127/433)	30 (69/229)	28 (58/204)	
-action	52 (225/433)	52 (119/229)	52 (106/204)	
**Exposed to passive smoking** (%, n/N)	26 (142/552)	28 (81/285)	23 (61/267)	.134*
**Drug use (NRT, Zyban®, Champix®) the week before baseline** (%, n/N)	52 (294/568)	53 (156/295)	51 (138/273)	.578*
**Snus use the week before baseline** (%, n/N)	7 (36/526)	9 (24/274)	5 (12/252)	.070*
**Other support** (%, n/N)**:**				
- none	28 (162/574)	28 (82/296)	29 (80/278)	.600*
- social	46 (264/574)	44 (131/296)	48 (133/278)	
- professional	11 (60/574)	12 (35/296)	9 (25/278)	
- social + professional	15 (88/574)	16 (48/296)	14 (40/278)	
**High level of client satisfaction at first contact** (%, n/N)^‡^	81 (447/554)	79 (228/288)	82 (219/266)	.346*
**Probability for being smokefree in one year** (1–10) (md, q_1−_q_3,_ N)	8, 7–10, 564	9, 7–10, 292	8, 7–10, 272	.209^†^
**Handle stress and depression successfully without smoking (**1–10) (md, q_1−_q_3,_ N)	7, 4–9, 564	7, 4–9, 293	7, 4–9, 271	.565^†^
**Will use pharmaceuticals if necessary** (1–10) (md, q_1−_q_3,_ N)	9, 5–10, 566	9, 5–10, 293	10, 5–10, 273	739^†^

*Statistical significant difference between proactive and reactive service tested with chi-square test.

^†^Statistical significant difference between proactive and reactive service tested with Mann–Whitney U-test.

^‡^Three questions: 1. The counsellor was understanding and sensitive, 2. The counsellor tried to understand my needs, 3. The counsellor showed respect for my own targets and decisions. Four response alternatives: much, rather, to some extent, not at all. Much for all the three questions was required for “much” and high level of client satisfaction in the analyses.

**Table 2:** Number and length of calls

	**Total**	**Proactive**	**Reactive**	***p*-value**
	**N = 586**	**n**_**pro**_ **= 303**	**n**_**re**_ **= 283**	
**Number of calls**				
Mean (SD)	3.2 (4.6)	4.3 (4.7)	2.1 (4.3)	< .001*
Median (q_1_-q_3_)	2.0 (1.0-3.0)	3.0 (2.0-5.0)	2.0 (1.0-2.0)	< .001^†^
Range	1-70	1-40	1-70	
1 call (%, n/N)	34 (198/586)	19 (57/303)	50 (141/283)	< .001^‡^
2 calls	31 (182/586)	27 (81/303)	36 (101/283)	
3 calls	11 (67/586)	15 (47/303)	7 (20/283)	
**Number of calls, 3 groups** (%, n/N)				
≤ 2	65 (380/586)	45 (138/303)	85 (242/283)	< .001^‡^
3-6	25 (145/586)	37 (111/303)	12 (34/283)	
≥ 7	10 (61/586)	18 (54/303)	3 (7/283)	
**Total length of calls** (minutes)				
Mean (SD)	47.9 (58.3)	60.1 (65.7)	34.8 (45.8)	< .001*
Median (q_1_-q_3_)	31.0 (20.7-52.0)	38.0 (24.0-71.0)	27.0 (18.0-38.0)	< .001^†^
Range	5-707	6-591	5-707	
**Length of first call** (minutes):				
Mean (SD)	25.1 (10.7)	25.2 (10.7)	25.0 (10.9)	.859*
Median (q_1_-q_3_)	23.0 (17.0-31.0)	23.0 (18.0-31.0)	23.0 (17.0-31.0)	.820^†^
Range	5-75	6-60	5-75	
**Length of second call** (minutes)	(N = 389)	(n = 246)	(n = 143)	
Mean (SD)	8.6 (9.5)	9.6 (9.7)	6.8 (8.9)	.004*
Median (q_1_-q_3_)	6.0 (1.0-13.0)	8.0 (2.0-14.3)	2.0 (0.0-12.0)	.001^†^
Range	0-61	0-61	0-40	

*Statistical significant difference between proactive and reactive service tested with *t*-test.

^†^Statistical significant difference between proactive and reactive service tested with Mann–Whitney *U*-test.

^‡^Statistical significant difference between proactive and reactive service tested with chi-square test.

There were no statistically significant differences in outcome between proactive and reactive service at the 12-month follow-up, in either point prevalence or continuous abstinence, or in either ITT or responder-only analyses. Of those who responded to the 12-month follow-up, 47% were point prevalence abstinent and 35% were continuously abstinent (Table 3).

**Table 3:** Abstinence at the 12-month follow-up

**% (n/N)**	**Total**	**Proactive**	**Reactive**	***p*-value**^**†**^
	**N = 586**	**n**_**pro**_ **= 303**	**n**_**re**_ **= 283**	
Response rate	59 (347/586)	55 (165/303)	64 (182/283)	.015
Point prevalence ITT*	27 (161/586)	26 (78/303)	29 (83/283)	.331
Point prevalence responder-only	47 (161/346)	47 (78/165)	46 (83/181)	.792
Continuous abstinence ITT*	21 (121/586)	20 (60/303)	22 (61/283)	.600
Continuous abstinence responder-only	35 (121/346)	36 (60/165)	34 (61/181)	.604

*Intention-to-treat. Abstinence measured in the whole study base, non-responders at 12-month follow-up treated as smokers.

^†^Statistical significant difference between proactive and reactive service tested with chi-square test.

The *predictive model* from the multivariable logistic regression analyses, which included only the variables known at baseline, showed that not smoking the week before baseline was the strongest predictor for both point prevalence (OR 3.2) and continuous abstinence (OR 3.7) at the 12-month follow-up. The ability to handle stress and depression without smoking was also a statistically significant predictor (upper part of Table 4). In the *descriptive model,* which included all variables until the 12-month follow-up, variables significant for point prevalence were i) not smoking the week before baseline, ii) the ability to handle stress and depression without smoking, iii) high level of client satisfaction at the first call, and iv) gender (female). Variables significant for continuous abstinence were i) not smoking the week before baseline, which had the strongest effect (OR 5.4), ii) baseline assessment of probability of being smoke-free in 1 year, and iii) not using NRT in the past week before follow-up (lower part of Table 4). The results of the univariable analyses for the two outcomes are presented in Additional file 1: Table S1 and Additional file 2: Table S2.

**Table 4:** **Multivariable logistic regression analyses**^**a **^**for point prevalence abstinence and 6-month continuous abstinence**

***Predictive model*** (variables at baseline related to being smoke-free at follow-up)				
	**Point prevalence abstinence**^**b**^		**6-month continuous abstinence**^**c**^	
**Variable**	**OR (95% CI)**	***p*-value**	**OR (95% CI)**	***p*-value**
**Service,** proactive vs. reactive (ref.)	0.7 (0.5-1.1)	.149	0.8 (0.5-1.3)	.423
**Gender,** men vs. women (ref.)	0.6 (0.4-1.0)	.061	0.7 (0.4-1.2)	.181
**Age**				
≤ 34 (ref.)	1.0	.846^f^	1.0	.748^f^
35-49	1.2 (0.7-2.1)	.578	1.3 (0.7-2.6)	.374
50-64	1.2 (0.7-2.1)	.552	1.2 (0.6-2.2)	.584
≥ 65	0.9 (0.5-1.9)	.884	1.0 (0.4-2.1)	.927
**Smoked in the week before baseline,** no vs. yes (ref.)	3.2 (2.1-4.9)	<.001	3.7 (2.3-5.9)	< .001
**Handle stress and depression without smoking** (baseline assessment, 1–10)	1.1 (1.04-1.2)	.003	1.2 (1.1-1.3)	< .001
***Descriptive model*** (all variables related to being smoke-free at follow-up)				
	**Point prevalence abstinence**^**d**^		**6-month continuous abstinence**^**e**^	
**Variable**	**OR (95% CI)**	***p*-value**	**OR (95% CI)**	***p*-value**
**Service,** proactive vs. reactive (ref.)	0.7 (0.5-1.1)	.163	0.7 (0.4-1.4)	.386
**Gender,** men vs. women (ref.)	0.6 (0.3-0.99)	.046	0.6 (0.3-1.1)	.111
**Age**				
≤ 34 (ref.)	1.0	.896^f^	1.0	.363^f^
35-49	1.1 (0.6-2.1)	.674	0.9 (0.4-2.2)	.896
50-64	1.1 (0.6-1.9)	.741	0.8 (0.4-1.8)	.612
≥ 65	0.9 (0.4-1.8)	.770	0.4 (0.1-1.2)	.111
**Smoked in the week before baseline,**	3.1 (2.0-4.9)	<.001	5.4 (3.0-9.7)	< .001
No vs. yes (ref.)				
**Handle stress and depression without smoking** (baseline assessment, 1–10)	1.1 (1.02-1.2)	.014	---	---
**Probability of being smoke-free in 1 year** (baseline assessment, 1–10)	---	---	1.3 (1.1-1.6)	< .001
**Level of client satisfaction at first contact,**	2.2 (1.2-4.0)	.013	---	---
High vs. other (ref.)				
**NRT use in the week before 12-month follow-up,** yes vs. no (ref.)	---	---	0.2 (0.1-0.5)	< .001

^a^Performed with service, gender and age forced into both models.

^b^N = 555, Nagelkerke R-Square 15.7%. Hosmer and Lemeshow test of goodness of fit, *p* = .057.

^c^N = 555, Nagelkerke R-Square 19.2%. Hosmer and Lemeshow test of goodness of fit, *p* = .265.

^d^N = 536, Nagelkerke R-Square 17.0%. Hosmer and Lemeshow test of goodness of fit, *p* = .365.

^e^N = 310, Nagelkerke R-Square 33.9%. Hosmer and Lemeshow test of goodness of fit, *p* = .621.

^f^*p*-value for the total effect of the variable with 3 df.

In *drop-out analyses*, we compared baseline characteristics of responders (n = 347) with non-responders (n = 239) to the 12-month follow-up. Responders were significantly more often in the reactive service, older, smoke-free at the first call, pharmaceutical users, and not exposed to second-hand smoke. They also smoked fewer cigarettes/day but had been smokers for a greater number of years. In a separate unpublished analysis of contacted responders and non-responders to the 12-month follow-up, we found that 54% (36/67) of responders were point prevalence abstinent 2.5-4 years after the first call, compared with 32% (14/44) of non-responders (*p* = .023, 74% response rate, data not shown).

## Discussion

### Main findings

Equal effectiveness was found in the proactive and reactive services at the SNTQ in the present study, and smoking status at baseline was the strongest predictor for abstinence at 12 months.

### Effectiveness

Almost half of those who responded to the 12-month had been smoke-free in the previous 7 days, and a third had been smoke-free for the past 6 months, in both the proactive and reactive service groups (Table 3). The effectiveness of the SNTQ has continuously improved over time, from below 30% to almost 40% in point prevalence among clients responding to the 12-month follow-up [19]. The present study indicates a further improvement. Even though international comparisons are difficult due to considerable differences in treatment protocols, organization, and techniques [8,10,13], the effectiveness in the present study compares favourably with most other reports, even those including NRT [2,5,8,10,11,20-22]. The specific definition used to define the study base at the SNTQ, yet, always has to be taken into consideration when comparing results to other quitlines. The equal effectiveness of the two services is somewhat surprising and in contrast to a number of international studies showing higher effectiveness for more intensive and proactive services [2,5]. However, it is in accordance with a recent study on the English quitline, which shows no additional effect of proactive counselling on abstinence after 6 months [11].

As Table 2 shows, there were significantly more calls, and consequently, significantly longer counselling duration, in the proactive service than in the reactive service. This would mean that the proactive service has an advantage, according to meta-analyses of telephone counselling, which show that increased intensity yields higher abstinence rates [2,5,10]. A difference in call volume is not obvious in practice, because participants using the reactive service can call many times and participants using the proactive service do not always accept or receive all scheduled calls [10,11,22].

Smoking status at baseline was the strongest predictor for abstinence at 12 months in the present study. Being smoke-free in the week prior to baseline increased the odds for abstinence by 3 to 5 times (Table 4). Nicotine dependence is usually simply measured as the number of cigarettes smoked per day, and is a frequently reported predictor in smoking cessation studies [2]. Unfortunately, the information on this variable from the data journal was invalid, and the question was not included in the baseline questionnaire. However, the baseline smoking status may be an appropriate alternative, because callers to a quitline are more prepared and motivated to quit than smokers in the general population and smokers in clinical trials [16,23], and therefore can be smoke-free relatively often at first contact. A confirmation was that different information sources were comparable; at first call 24% reported being tobacco-free and 27% reported no (0) smoked cigarettes/day, and at baseline 28% reported being smoke-free in the past week, with no differences between the proactive and reactive services.

A meta-analysis of 22 studies finds that proactive telephone counselling has a significant adjunct effect to other minimal interventions for younger, male, light-smoking participants [24]. None of the study characteristics related to intervention process, such as number of calls and minutes per call, significantly explained variations in outcome. An implication of that analysis is the need to focus on participants as much as on intervention processes to obtain more effective interventions. The rationale for the present study was the need to make the best use of limited resources, and one way would be to provide treatment related to individual performance. We found no differences between the two services for gender or different age groups. This contrasts with the previously mentioned, but non-randomized SNTQ study where the proactive service is found to be more effective in women than in men [16].

### Possible explanations

There are a number of possible explanations for the observed equal effectiveness of the proactive and reactive services in the present study. The SNTQ already had a relatively high success rate and this may be one possible reason for the lack of difference between the service alternatives. Everyone calling the SNTQ has initiated a call in the first place, indicating that their motivation to quit may be higher than that of the average smoker trying to quit or of smokers recruited to cessation studies. Approximately every fourth caller were smoke-free at baseline in both services, and these people probably have the highest motivation which is supported by the finding that the “smoke-free at baseline” variable was the most important predictive factor for abstinence after 1 year in our regression analyses (Table 4).

The length of the first call was equal in both services (Table 2), and it is certainly possible that the high quality of the initial interviews may have been a factor in reducing the difference between the services. During the time of the present study high quality MI was delivered in both services according to laboratory coding. Adding high quality MI to the SNTQ treatment protocol is found to significantly enhance the already relatively high treatment outcome by approximately 5%, in a previous controlled clinical trial [19]. The present results suggest that clients reporting a high level of satisfaction at first contact were twice as likely to be point prevalent abstinent after 1 year (Table 4). There was no significant difference between the services regarding level of client satisfaction.

The reactive service at the SNTQ has, to some extent, a proactive element in it because all clients are followed up after 12 months, and the effect of the 12-month follow-up *per se* is unknown. There was a higher response rate at the 12-month follow-up in the reactive service group than the proactive service group. This difference in response rate is not yet explained and is somewhat surprising, because recruitment and follow-up routines were the same for both groups. If anything, these differences may underestimate the effectiveness of the proactive service compared with the reactive service, because responders may be more likely to be smoke-free at follow-up as demonstrated in the higher abstinence rates in responder-only compared with ITT-analyses (Table 3).

### NRT and snus

The long-term effectiveness of NRT seen in clinical trials has been questioned when used in real-world settings and in general populations [11,25,26]. In the present study, we found no effect of NRT use between the first call and the 12-month follow-up in the multivariable analyses, which is consistent with results from the English national quitline study [11]. On the contrary, those who used NRT in the week before the follow-up were significantly less likely to be continuously abstinent (Table 3). A possible explanation can be that these NRT users were in a new or repeated quit attempt at the time of follow-up, since it concerned continuous abstinence but not point prevalence abstinence.

Snus is debated as a means for smoking cessation and seems to play a different role for smokers who quit with professional support than it does for self-quitters [16,27-29]. In the present clinical setting, only 7% of the participants used snus the week before baseline and 7% used it the week before 12-month follow-up, and we found no effect of snus use on abstinence in the multivariable analyses. This is consistent with previous data from the SNTQ [16].

### Methodological matters, strengths and limitations

Quitline trials are, in most cases, effectiveness trials because they are conducted within the context of operating quitlines, and therefore under relatively real-world conditions [5,13]. The present study was the first attempt at the SNTQ to randomize clients within the normal running activity to assess if offering proactivity would enhance quit rates. As randomization was performed on even and odd dates, thus at the time for the client’s first call, the intervention had started before the client was included in the study, as a returned baseline questionnaire is required for inclusion in the SNTQ study base. The definition of the SNTQ study base has remained constant from the start in 1998, to enable comparisons of changes in outcome with changes in the treatment protocol over time. Whereas this may be a strength for the internal development of the SNTQ service, it may be a potential problem when comparing SNTQ results with other smoking cessation services.

Under real-world conditions, many practical factors such as invalid phone numbers and missed appointments influence the intervention actually delivered. Furthermore, in a study such as the present, even human factors such as forgetfulness, specific wishes, and the considerations of callers and counsellors can end in protocol violation. Thirteen per cent of those randomized to reactive service actually received proactive service, a figure that ought to be zero, and those clients had to be excluded from the study. A possible explanation is the randomization procedure to proactive service on even dates and to reactive service on odd dates, which required the counsellors to remember and then strictly abide by what was stated in the protocol.

The equal effectiveness of both services in the ITT- as well as the responder-only analyses is a strength of the present study. The study was terminated before reaching the required number of clients according to the power calculation. A part-time analysis was entered in the syllabus and this analysis revealed no additional effect of the proactive service over the reactive, thus it was decided to discontinue new recruitment to the study.

A limitation regarding validity is the self-report of abstinence, which may overestimate quit rates. However, self-reports are considered accurate in most smoking cessation studies and are rarely differential across intervention conditions. Biochemical verification is not required and may not be desirable in studies where the optimal data collection methods are through mail, telephone, or the Internet [30]. The tradition in evaluating smoking cessation programmes is to treat non-responders as smokers. It is a conservative approach that will not overestimate treatment effects. However, a previous study of non-responders of the SNTQ indicates that it may also underestimate the true treatment effect [31]. The response rate of 59% at the 12-month follow-up is a matter of concern. Although follow-up procedures have not changed, SNTQ response rates have decreased over time from about 70% to 60% [16,19]. Even if relatively normal in studies like this [8], there is a possibility for bias due to differential loss to follow-up, especially in light of the described differences between responders and non-responders.

## Conclusion

To optimize resource utilization at the SNTQ, the reactive service may be preferred as the first treatment of choice. However, more research is needed to assess whether the proactive service may be favourable for subgroups of clients.

## Competing interests

The authors declare that they have no competing interests.

## Authors’ contributions

EN developed and managed the database, carried out the analyses, prepared the manuscript and acted as corresponding author. JÖ supervised the statistical analyses and participated in the writing of the paper. ÁRH constructed the study and formulated the hypothesis, contributed with tobacco cessation expertise and participated in the supervision of the study and writing of the paper. All authors have approved the final manuscript.

## Authors’ information

Eva Nohlert, PhD, DDS; John Öhrvik, PhD, professor in Biostatistics; Ásgeir R Helgason, PhD, associate professor in Psychology.

## Supplementary Material

Additional file 1: Table S1 Univariable logistic regression analyses for point prevalence abstinence. Click here for file

Additional file 2: Table S2 Univariable logistic regression analyses for 6-month continuous abstinence. Click here for file

## References

[B1] WHO WHO Report On The Global Tobacco Epidemic, 2011 2011 Geneva: World Health Organization (WHO)

[B2] Fiore MC Jaén CR Baker TB Bailey WC Benowitz NL Curry SJ Faith Dorfman S Froelicher ES Goldstein MG Healton CG Nez Henderson P Heyman RB Koh HK Kottke TE Lando HA Mecklenburg RE Mermelstein RJ Dolan Mullen P Orleans CT Robinson L Stitzer ML Tommasello AC Villejo L Wewers M-E Treating Tobacco Use and Dependence: 2008 Update. Clinical Practice Guideline 2008 Rockville, MD: U.S. Department of Health and Human Service. Public Health Service

[B3] Helgason AR Lund KE Adolfsson J Axelsson S Tobacco prevention in Swedish dental care Community Dent Oral Epidemiol 2003 31 5 378 385 10.1034/j.1600-0528.2003.00111.x 14667009

[B4] Stead M Angus K Holme I Cohen D Tait G Factors influencing European GPs’ engagement in smoking cessation: a multi-country literature review Br J Gen Pract 2009 59 566 682 690 10.3399/bjgp09X454007 19674514 PMC2734357

[B5] Stead LF Perera R Lancaster T A systematic review of interventions for smokers who contact quitlines Tob Control 2007 16 Suppl 1 i3 i8 18048627 10.1136/tc.2006.019737 PMC2598525

[B6] Borland R Segan CJ The potential of quitlines to increase smoking cessation Drug Alcohol Rev 2006 25 1 73 78 10.1080/09595230500459537 16492579

[B7] Smith SS Keller PA Kobinsky KH Baker TB Fraser DL Bush T Magnusson B Zbikowski SM McAfee TA Fiore MC Enhancing tobacco quitline effectiveness: identifying a superior pharmacotherapy adjuvant Nicotine Tob Res 2013 15 3 718 728 10.1093/ntr/nts186 22992296 PMC3611992

[B8] Willemsen MC van der Meer RM Bor S Description, Effectiveness, and Client Satisfaction of 9 European Quitlines: Results of the European Smoking Cessation Helplines Evaluation Project (ESCHER) 2008 Den Haag: STIVORO

[B9] Stead LF Hartmann-Boyce J Perera R Lancaster T Telephone counselling for smoking cessation Cochrane Database Syst Rev 2013 8 CD002850 10.1002/14651858.CD002850.pub3 23934971

[B10] Stead LF Perera R Lancaster T Telephone counselling for smoking cessation Cochrane Database Syst Rev 2006 3 CD002850 10.1002/14651858.CD002850.pub2 16855992

[B11] Ferguson J Docherty G Bauld L Lewis S Lorgelly P Boyd KA McEwen A Coleman T Effect of offering different levels of support and free nicotine replacement therapy via an English national telephone quitline: randomised controlled trial BMJ 2012 344 e1696 10.1136/bmj.e1696 22446739 PMC3311694

[B12] Gilbert H Sutton S Evaluating the effectiveness of proactive telephone counselling for smoking cessation in a randomized controlled trial Addiction 2006 101 4 590 598 10.1111/j.1360-0443.2006.01398.x 16548938

[B13] Lichtenstein E Zhu SH Tedeschi GJ Smoking cessation quitlines: an underrecognized intervention success story Am Psychol 2010 65 4 252 261 20455619 10.1037/a0018598 PMC3169380

[B14] The National Swedish Board of Health and Welfare and Swedish National Institute of Public Health Public health in Sweden - Annual report 2013 2013 Stockholm: The National Swedish Board of Health and Welfare and Swedish National Institute of Public Health

[B15] Swedish National Institute of Public Health Tobacco and Weaning 2009 Östersund: Swedish National Institute of Public Health

[B16] Helgason AR Tomson T Lund KE Galanti R Ahnve S Gilljam H Factors related to abstinence in a telephone helpline for smoking cessation Eur J Public Health 2004 14 3 306 310 10.1093/eurpub/14.3.306 15369039

[B17] Tomson T Helgason AR Gilljam H Quitline in smoking cessation: a cost-effectiveness analysis Int J Technol Assess Health Care 2004 20 4 469 474 15609797 10.1017/s0266462304001370

[B18] Hosmer DW Lemeshow S Applied logistic regression 1989 New York: Wiley

[B19] Lindqvist H Forsberg LG Forsberg L Rosendahl I Enebrink P Helgason AR Motivational Interviewing in an ordinary clinical setting: a controlled clinical trial at the Swedish National Tobacco Quitline Addict Behav 2013 38 7 2321 2324 10.1016/j.addbeh.2013.03.002 23584193

[B20] Segan CJ Borland R Does extended telephone callback counselling prevent smoking relapse? Health Educ Res 2011 26 2 336 347 10.1093/her/cyr009 21402601

[B21] An LC Schillo BA Kavanaugh AM Lachter RB Luxenberg MG Wendling AH Joseph AM Increased reach and effectiveness of a statewide tobacco quitline after the addition of access to free nicotine replacement therapy Tob Control 2006 15 4 286 293 10.1136/tc.2005.014555 16885577 PMC2563594

[B22] Carlin-Menter S Cummings KM Celestino P Hyland A Mahoney MC Willett J Juster HR Does offering more support calls to smokers influence quit success? J Public Health Manag Pract 2011 17 3 E9 E15 10.1097/PHH.0b013e318208e730 21464680

[B23] Etter JF Perneger T Ronchi A Distributions of smokers by stage: International comparison and association with smoking prevalence Prev Med 1997 26 580 585 10.1006/pmed.1997.0179 9245682

[B24] Pan W Proactive telephone counseling as an adjunct to minimal intervention for smoking cessation: a meta-analysis Health Educ Res 2006 21 3 416 427 10.1093/her/cyl040 16740673

[B25] Alberg AJ Patnaik JL May JW Hoffman SC Gitchelle J Comstock GW Helzlsouer KJ Nicotine replacement therapy use among a cohort of smokers J Addict Dis 2005 24 1 101 113 10.1300/J069v24n01_09 15774414

[B26] Alpert HR Connolly GN Biener L A prospective cohort study challenging the effectiveness of population-based medical intervention for smoking cessation Tob Control 2013 22 1 32 37 10.1136/tobaccocontrol-2011-050129 22234781

[B27] Gilljam H Galanti R Role of *snus* (oral moist snuff) in smoking cessation and smoking reduction in Sweden Addiction 2003 98 1183 1189 10.1046/j.1360-0443.2003.00379.x 12930201

[B28] Lund KE Scheffels J McNeill A The association between use of snus and quit rates for smoking: results from seven Norwegian cross-sectional studies Addiction 2011 106 1 162 167 10.1111/j.1360-0443.2010.03122.x 20883459 PMC3021722

[B29] Fagerstrom K Rutqvist LE Hughes JR Snus as a smoking cessation aid: a randomized placebo-controlled trial Nicotine Tob Res 2012 14 3 306 312 10.1093/ntr/ntr214 21994343

[B30] Society for Research on Nicotine and Tobacco Subcommittee on Biochemical Verification Biochemical verification of tobacco use and cessation Nicotine Tob Res 2002 4 2 149 159 10.1080/14622200210123581 12028847

[B31] Tomson T Bjornstrom C Gilljam H Helgason A Are non-responders in a quitline evaluation more likely to be smokers? BMC Public Health 2005 5 1 52 10.1186/1471-2458-5-52 15910682 PMC1173115

