# Supplementary material for: Effectiveness of proactive and reactive services at the Swedish National Tobacco Quitline in a randomized trial
Source: Tob Induc Dis. 2014 Jun 3;12(1):9. doi: 10.1186/1617-9625-12-9 (PMC4059482; doi:10.1186/1617-9625-12-9)
Supplement: Additional file 1: Table S1 — Univariable logistic regression analyses for point prevalence abstinence. [file 1617-9625-12-9-S1.docx]

Additional file 1: Table S1. Univariable logistic regression analyses for point prevalence abstinence

| **Variable** | **n/N*** | **OR (95% CI for OR)** | **p-value** |
| --- | --- | --- | --- |
| Service; proactive vs. reactive (ref) | 303/586 vs. 283/586 | 0.83 (0.58-1.20) | .331 |
| Gender; men vs. women (ref) | 129/586 vs. 457/586 | 0.60 (0.37-0.97) | .036 |
| Age; - ≤ 34 (ref) - 35-49 - 50-64 - ≥ 65 | 115/576 142/576 223/576 96/576 | 1.0 1.31 (0.75-2.28) 1.36 (0.82-2.28) 0.87 (0.46-1.66) | .338 .350 .236 .672 |
| Stages-of-change (data journal); -precontemplation/don´t know (ref) -contemplation -preparation -action -maintenance | 108/585 96/585 212/585 162/585 7/585 | 1.0 0.59 (0.27-1.28) 1.52 (0.87-2.68) 2.78 (1.57-4.91) 24.86 (2.84-217.70) | <.001 .182 .144 <.001 .004 |
| Tobacco free at first call (data journal); yes vs. no (ref) | 143/586 vs. 443/586 | 3.56 (2.38-5.31) | <.001 |
| Number of smoked cig/day (data journal - Obs difficult to interpret) | md=10, q_1_=0, q_3_=20, N=482 | 0.94 (0.91-0.96) | <.001 |
| *Variables from baseline questionnaire* | | | |
| Number of years of education | md=12, q_1_=10, q_3_=13, N=570 | 1.04 (0.98-1.11) | .174 |
| Number of years smoked before baseline | md=33, q_1_=18, q_3_=40, N=552 | 1.00 (0.99-1.01) | .990 |
| Smoking the week before baseline; - daily (ref) - not daily - none | 338/583 84/583 161/583 | 1.0 1.07 (0.59-1.94) 4.05 (2.68-6.10) | <.001 .835 <.001 |
| Smoking the week before baseline; no vs. yes (ref) | 161/583 vs. 422/583 | 3.99 (2.70-5.91) | <.001 |
| Time since last puff; - 0-7 days (ref) - > 7 days but < 6 months - ≥ 6 months | 414/559 137/559 8/559 | 1.0 3.25 (2.15-4.89) 11.11 (2.21-56.02) | <.001 <.001 .004 |
| Passive smoking at baseline;  -almost every day (ref) -some time a week -some time a month -never/almost never | 55/552 56/552 31/552 410/552 | 1.0 1.21 (0.49-2.99) 0.96 (0.32-2.91) 1.71 (0.86-3.43) | .232 .681 .942 .128 |
| Passive smoking at baseline; not exposed vs. exposed (ref) | 410/552 vs. 142/552 | 1.60 (1.01-2.52) | .043 |
| Stages-of-change (baseline); -precontemplation/contemplation (ref) -preparation -action | 81/433 127/433 225/433 | 1.0 2.13 (0.91-5.00) 2.88 (1.31-6.36) | .028 .084 .009 |
| Drug use (NRT, Zyban®, Champix®) the week before baseline;  yes vs. no (ref) | 294/568 vs. 274/568 | 1.76 (1.21-2.56) | .003 |
| Snus use – present or ever;  - daily/almost daily - intermittent (off and on) - none | 41/527 34/527 452/527 | 1.0 0.92 (0.30-2.80) 1.41 (0.65-3.03) | .453 .886 .385 |
| Snus use the week before baseline;  - daily (ref) - not daily - none | 24/526 12/526 490/526 | 1.0 0.45 (0.05-4.59) 1.98 (0.67-5.90) | .181 .504 .220 |
| Snus use the week before baseline; no vs. yes (ref) | 490/526 vs. 36/526 | 2.45 (0.94-6.44) | .068 |
| Other support at baseline: - none (ref) - social - professional - social+professional | 162/574 264/574 60/574 88/574 | 1.0 1.58 (0.99-2.52) 1.42 (0.71-2.83) 2.23 (1.25-3.98) | .052 .054 .317 .006 |
| Level of client satisfaction at first contact (baseline, 3 questions)**^†^**; high vs. everything else (ref) | 447/554 vs. 107/554 | 1.96 (1.15-3.35) | .014 |
| Probability for being smokefree in one year (baseline, 1-10) | md=8, q_1_=7, q_3_=10, N=564 | 1.22 (1.10-1.35) | <.001 |
| Handle stress and depression successfully without smoking  (baseline, 1-10) | md=7, q_1_=4, q_3_=9, N=564 | 1.21 (1.12-1.30) | <.001 |
| Will use pharmaceuticals if necessary (baseline,1-10) | md=9, q_1_=5, q_3_=10, N=566 | 0.99 (0.94-1.05) | .785 |
| Smoke when feel depressed (baseline); - always/almost always (ref) - sometimes - seldom - never | 363/555 144/555 36/555 12/555 | 1.0 0.97 (0.63-1.49) 0.84 (0.38-1.85) 1.26 (0.37-4.28) | .950 .893 .668 .709 |
| *Variables from 12-month follow-up questionnaire* | | | |
| Number of years smoked (12-month follow-up); - > 20 (ref) - 15-20 - 10-15 - 5-10 - 1-5 | 236/337 43/337 30/337 21/337 7/337 | 1.0 1.00 (0.52-1.91) 1.50 (0.70-3.22) 1.26 (0.51-3.08) 0.19 (0.02-1.61) | .442 .990 .301 .612 .128 |
| Max length of earlier smoke-free period, number of months | md=12, q_1_=3, q_3_=17, N=312 | 1.00 (1.00-1.01) | .619 |
| Passive smoking at 12-month follow-up;  -almost every day (ref) -some time a week -some time a month -never/almost never | 36/335 18/335 12/335 269/335 | 1.0 1.82 (0.57-5.85) 2.27 (0.60-8.64) 2.19 (1.04-4.63) | .230 .316 .228 .040 |
| Passive smoking at 12-month follow-up; not exposed vs. exposed (ref) | 269/335 vs. 66/335 | 1.58 (0.91-2.74) | .104 |
| NRT use the week before 12-month follow-up; yes vs. no (ref) | 72/331 vs. 259/331 | 0.97 (0.57-1.63) | .894 |
| NRT**^‡^** use between first call and 12-month follow-up; - none (ref) - < 5 weeks - ≥ 5 weeks | 133/320 107/320 80/320 | 1.0 0.50 (0.29-0.85) 1.27 (0.73-2.21) | .005 .010 .406 |
| Drug use between first call and 12-month follow-up; - none (ref) - NRT - Zyban® - Champix® - NRT/Zyban®/Champix® in different combinations - other ( Zonnic) | 74/320 167/320 18/320 35/320 22/320 4/320 | 1.0 0.73 (0.42-1.27) 0.72 (0.25-2.02) 1.07 (0.48-2.39) 0.34 (0.12-0.95) 0.30 (0.03-3.01) | .310 .264 .531 .877 .041 .306 |
| Drug use (NRT, Zyban®, Champix®) between first call and 12-month follow-up, yes vs. no (ref) | 242/316 vs. 74/316 | 0.72 (0.43-1.22) | .224 |
| Zyban® use between first call and 12-month follow-up; - none (ref) - < 7 weeks - ≥ 7 weeks | 290/320 16/320 14/320 | 1.0 0.51 (0.17-1.50) 0.84 (0.28-2.47) | .454 .219 .748 |
| Champix® use between first call and 12-month follow-up; - none (ref) - < 12 weeks - ≥ 12 weeks | 273/320 34/320 13/320 | 1.0 1.04 (0.51-2.12) 1.00 (0.33-3.05) | .995 .921 1.000 |
| Snus use the week before 12-month follow-up;  - daily (ref) - not daily - none | 11/268 9/268 248/268 | 1.0 0.33 (0.03-3.93) 2.46 (0.64-9.49) | .078 .383 .191 |
| Snus use the week before 12-month follow up; no vs. yes (ref) | 248/268 vs. 20/268 | 3.69 (1.20-11.35) | .023 |
| Other support at 12-month follow-up;  - none (ref) - social - professional - social+professional | 75/328 153/328 37/328 63/328 | 1.0 2.25 (1.27-4.00) 1.70 (0.76-3.80) 2.20 (1.10-4.38) | .040 .006 .197 .025 |
| Grading of support/help from SNTQ (1-10) | md=7, q_1_=4, q_3_=9, N=331 | 1.18 (1.09-1.28) | <.001 |
| Effect on motivation to quit from SNTQ (independent of success in quitting);  - much - rather - in some degree - not at all | 107/331 66/331 106/331 52/331 | 1.0 0.53 (0.29-1.00) 0.58 (0.34-1.00) 0.38 (0.19-0.77) | .029 .048 .048 .006 |
| Compliance at 12-month follow-up (1-10) | md=6, q_1_=4, q_3_=8, N=321 | 1.14 (1.05-1.23) | .002 |
| Abnormally stressed or depressed since first contact; yes vs. no (ref) | 211/332 vs. 121/332 | 1.10 (0.70-1.73) | .672 |
| *Number and length of calls* | | | |
| Total number of calls | md=2, q_1_=1, q_3_=3, N=586 | 1.05 (1.01-1.09) | .028 |
| Total number of calls, 3 groups; - ≤ 2 (ref) - 3-6 - ≥ 7 | 380/586 145/586 61/586 | 1.0 1.24 (0.81-1.90) 2.11 (1.21–3.70) | .030 .323 .009 |
| Total length of calls (minutes) | md=31, q_1_=21, q_3_=52, N=586 | 1.004 (1.001-1.01) | .008 |
| Total length of calls (hours) | md=0.52, q_1_=0.35, q_3_=0.87, N=586 | 1.29 (1.07-1.56) | .008 |
| Length of call 1 (minutes) | md=23, q_1_=17, q_3_=31, N=586 | 1.00 (0.98-1.01) | .855 |
| Length of call 2 | md=6, q_1_=1, q_3_=13, N=389 | 1.03 (1.01-1.05) | .014 |
| Length of call 3 | md=10, q_1_=5, q_3_=17, N=206 | 1.01 (0.98-1.05) | .505 |
| Length of call 4 | md=9, q_1_=5, q_3_=17, N=139 | 1.01 (0.97-1.04) | .732 |
| Length of call 5 | md=8, q_1_=4, q_3_=16, N=104 | 1.03 (0.98-1.09) | .183 |
| Number of days between call 1 and 2 | md=13, q_1_=7, q_3_=23, N=389 | 1.00 (0.99-1.01) | .946 |
| Number of days between call 1 and 3 | md=18, q_1_=13, q_3_=29, N=206 | 1.00 (0.99-1.01) | .993 |
| Number of days between call 1 and 4 | md=30, q_1_=20, q_3_=50, N=139 | 1.00 (0.99-1.01) | .454 |
| Number of days between call 1 and 5 | md=42, q_1_=28, q_3_=67, N=104 | 0.99 (0.98-1.00) | .181 |

^*^ n=number in category, N=total number in analysis.
**^†^** Three questions: 1. The counsellor was understanding and sensitive, 2. The counsellor tried to understand my needs, 3. The counsellor showed respect for my own targets and decisions. Four response alternatives: much, rather, to some extent, not at all. Much for all the three questions was required for “much” in the analysis.
**^‡^** Max number of weeks for any preparation.
