# Supplementary material for: Effectiveness of proactive and reactive services at the Swedish National Tobacco Quitline in a randomized trial
Source: Tob Induc Dis. 2014 Jun 3;12(1):9. doi: 10.1186/1617-9625-12-9 (PMC4059482; doi:10.1186/1617-9625-12-9)
Supplement: Additional file 2: Table S2 — Univariable logistic regression analyses for 6-month continuous abstinence. [file 1617-9625-12-9-S2.docx]

Additional file 2: Table S2. Univariable logistic regression analyses for 6-month continuous abstinence

| **Variable** | **n/N*** | **OR (95% CI for OR)** | **p-value** |
| --- | --- | --- | --- |
| Service; proactive vs. reactive (ref) | 303/586 vs. 283/586 | 0.90 (0.60-1.34) | .600 |
| Gender; men vs. women (ref) | 129/586 vs. 457/586 | 0.65 (0.38-1.09) | .104 |
| Age; - ≤ 34 (ref) - 35-49 - 50-64 - ≥ 65 | 115/576 142/576 223/576 96/576 | 1.0 1.49 (0.81-2.77) 1.37 (0.77-2.44) 0.88 (0.42-1.83) | .313 .201 .281 .731 |
| Stages-of-change (data journal); -precontemplation/don´t know (ref) -contemplation -preparation -action -maintenance | 108/585 96/585 212/585 162/585 7/585 | 1.0 0.56 (0.23-1.39) 1.40 (0.73-2.67) 3.01 (1.59-5.70) 37.20 (4.18-331.10) | <.001 .215 .310 .001 .001 |
| Tobacco free at first call (data journal); yes vs. no (ref) | 143/586 vs. 443/586 | 4.32 (2.81-6.62) | <.001 |
| Number of smoked cig/day (data journal – Obs difficult to interpret) | md=10, q_1_=0, q_3_=20, N=482 | 0.93 (0.91-0.96) | <.001 |
| *Variables from baseline questionnaire* | | | |
| Number of years of education | md=12, q_1_=10, q_3_=13, N=570 | 1.04 (0.97-1.11) | .275 |
| Number of years smoked (baseline) | md=33, q_1_=18, q_3_=40, N=552 | 1.01 (0.99-1.02) | .417 |
| Smoking the week before baseline; - daily (ref) - not daily - none | 338/583 84/583 161/583 | 1.0 0.80 (0.37-1.71) 4.89 (3.13-7.63) | <.001 .569 <.001 |
| Smoking the week before baseline; no vs. yes (ref) | 161/583 vs. 422/583 | 5.09 (3.33-7.79) | <.001 |
| Time since last puff; - 0-7 days (ref) - > 7 days but < 6 months - ≥ 6 months | 414/559 137/559 8/559 | 1.0 4.04 (2.60-6.26) 18.05 (3.56-91.57) | <.001 <.001  <.001 |
| Passive smoking at baseline;  -almost every day (ref) -some time a week -some time a month -never/almost never | 55/552 56/552 31/552 410/552 | 1.0 0.82 (0.26-2.63) 1.32 (0.38-4.57) 2.07 (0.91-4.72) | .066 .742 .663 .085 |
| Passive smoking at baseline; not exposed vs. exposed (ref) | 410/552 vs. 142/552 | 2.08 (1.21-3.58) | .009 |
| Stages-of-change (baseline); -precontemplation/contemplation (ref) -preparation -action | 81/433 127/433 225/433 | 1.0 1.55 (0.57-4.21) 2.54 (1.03-6.26) | .071 .391 .043 |
| Drug use (NRT, Zyban®, Champix®) the week before baseline;  yes vs. no (ref) | 294/568 vs. 274/568 | 1.57 (1.04-2.36) | .033 |
| Snus use – present or ever; - daily/almost daily - intermittent (off and on) - none | 41/527 34/527 452/527 | 1.0 0.78 (0.20-3.02) 1.64 (0.67-4.00) | .236 .716 .281 |
| Snus use the week before baseline;  - daily (ref) - not daily - none | 24/526 12/526 490/526 | 1.0 0.00 (0.00-) 3.07 (0.71-13.28) | .323 .999 .133 |
| Snus use the week before baseline; no vs. yes (ref) | 490/526 vs. 36/526 | 4.75 (1.12-20.09) | .034 |
| Other support at baseline: - none (ref) - social - professional - social+professional | 162/574 264/574 60/574 88/574 | 1.0 1.35 (0.80-2.25) 1.31 (0.61-2.79) 2.31 (1.25-4.29) | .062 .259 .488 .008 |
| Level of client satisfaction at first contact (baseline, 3 questions)**^†^**; high vs. everything else (ref) | 447/554 vs. 107/554 | 2.14 (1.15-3.98) | .016 |
| Probability for being smokefree in one year (baseline, 1-10) | md=8, q_1_=7, q_3_=10, N=564 | 1.34 (1.18-1.52) | <.001 |
| Handle stress and depression successfully without smoking  (baseline, 1-10) | md=7, q_1_=4, q_3_=9, N=564 | 1.28 (1.17-1.39) | <.001 |
| Will use pharmaceuticals if necessary (baseline,1-10) | md=9, q_1_=5, q_3_=10, N=566 | 0.98 (0.92-1.04) | .481 |
| Smoke when feel depressed (baseline); - always/almost always (ref) - sometimes - seldom - never | 363/555 144/555 36/555 12/555 | 1.0 0.82 (0.50-1.33) 1.01 (0.44-2.30) 1.18 (0.31-4.46) | .852 .414 .980 .808 |
| *Variables from 12-month follow-up questionnaire* | | | |
| Number of years smoked (12-month follow-up); - > 20 (ref) - 15-20 - 10-15 - 5-10 - 1-5 | 236/337 43/337 30/337 21/337 7/337 | 1.0 0.63 (0.31-1.29) 1.08 (0.50-2.35) 0.65 (0.24-1.73) 0.27 (0.03-2.28) | .456 .203 .843 .388 .229 |
| Max length of earlier smoke-free period, number of months | md=12, q_1_=3, q_3_=17, N=312 | 1.00 (1.00-1.01) | .654 |
| Passive smoking at 12-month follow-up;  -almost every day (ref) -some time a week -some time a month -never/almost never | 36/335 18/335 12/335 269/335 | 1.0 2.50 (0.67-9.31) 2.50 (0.57-11.05) 3.10 (1.25-7.71) | .109 .172 .227 .015 |
| Passive smoking at 12-month follow-up; not exposed vs. exposed (ref) | 269/335 vs. 66/335 | 1.94 (1.05-3.58) | .035 |
| NRT use the week before 12-month follow-up; yes vs. no (ref) | 72/331 vs. 259/331 | 0.37 (0.20-0.70) | .002 |
| NRT**^‡^** use between first call and 12-month follow-up; - none (ref) - < 5 weeks - ≥ 5 weeks | 133/320 107/320 80/320 | 1.0 0.40 (0.23-0.71) 1.14 (0.65-1.99) | .002 .002 .652 |
| Drug use between first call and 12-month follow-up; - none (ref) - NRT - Zyban® - Champix® - NRT/Zyban®/Champix® in different combinations - other (Zonnic) | 74/320 167/320 18/320 35/320 22/320 4/320^§^ | 1.0 0.70 (0.40-1.23) 1.17 (0.41-3.32) 1.23 (0.55-2.78) 0.33 (0.10-1.06) - | .268 .219 .763 .610 .062 - |
| Drug use (NRT, Zyban®, Champix®) between first call and 12-month follow-up, yes vs. no (ref) | 242/316 vs. 74/316 | 0.75 (0.44-1.28) | .296 |
| Zyban® use between first call and 12-month follow-up; - none (ref) - < 7 weeks - ≥ 7 weeks | 290/320 16/320 14/320 | 1.0 0.84 (0.28-2.48) 1.02 (0.33-3.14) | .949 .749 .967 |
| Champix® use between first call and 12-month follow-up; - none (ref) - < 12 weeks - ≥ 12 weeks | 273/320 34/320 13/320 | 1.0 1.18 (0.57-2.46) 1.19 (0.38-3.74) | .877 .661 .766 |
| Snus use the week before 12-month follow-up;  - daily (ref) - not daily - none | 11/268 9/268 248/268 | 1.0 0.00 (0.00-) 2.65 (0.56-12.55) | .469 .999 .218 |
| Snus use the week before 12-month follow up; no vs. yes (ref) | 248/268 vs. 20/268 | 5.31 (1.20-23.40) | .027 |
| Other support at 12-month follow-up;  - none (ref) - social - professional - social+professional | 75/328 153/328 37/328 63/328 | 1.0 1.49 (0.81-2.71) 1.57 (0.68-3.60) 2.06 (1.01-4.18) | .257 .198 .292 .046 |
| Grading of support/help from SNTQ (1-10) | md=7, q_1_=4, q_3_=9, N=331 | 1.14 (1.05-1.24) | .002 |
| Effect on motivation to quit from SNTQ (independent of success in quitting);  - much - rather - in some degree - not at all | 107/331 66/331 106/331 52/331 | 1.0 0.71 (0.38-1.34) 0.60 (0.34-1.05) 0.54 (0.26-1.09) | .213 .289 .074 .087 |
| Compliance at 12-month follow-up (1-10) | md=6, q_1_=4, q_3_=8, N=321 | 1.11 (1.01-1.20) | .022 |
| Abnormally stressed or depressed since first contact; yes vs. no (ref) | 211/332 vs. 121/332 | 0.91 (0.57-1.45) | .698 |
| *Number and length of calls* | | | |
| Total number of calls | md=2, q_1_=1, q_3_=3, N=586 | 1.00 (0.96-1.05) | .975 |
| Total number of calls, 3 groups; - ≤ 2 (ref) - 3-6 - ≥ 7 | 380/586 145/586 61/586 | 1.0 1.36 (0.85-2.16) 1.71 (0.92-3.17) | .154 .197 .088 |
| Total length of calls (minutes) | md=31, q_1_=21, q_3_=52, N=586 | 1.00 (1.00-1.00) | .697 |
| Total length of calls (hours) | md=0.52, q_1_=0.35, q_3_=0.87, N=586 | 1.04 (0.86-1.26) | .697 |
| Length of call 1 (minutes) | md=23, q_1_=17, q_3_=31, N=586 | 1.00 (0.98-1.01) | .698 |
| Length of call 2 | md=6, q_1_=1, q_3_=13, N=389 | 1.02 (1.00-1.05) | .055 |
| Length of call 3 | md=10, q_1_=5, q_3_=17, N=206 | 1.01 (0.97-1.05) | .632 |
| Length of call 4 | md=9, q_1_=5, q_3_=17, N=139 | 1.01 (0.97-1.05) | .616 |
| Length of call 5 | md=8, q_1_=4, q_3_=16, N=104 | 1.02 (0.97-1.07) | .506 |
| Number of days between call 1 and 2 | md=13, q_1_=7, q_3_=23, N=389 | 1.00 (0.99-1.01) | .765 |
| Number of days between call 1 and 3 | md=18, q_1_=13, q_3_=29, N=206 | 1.00 (1.00-1.01) | .450 |
| Number of days between call 1 and 4 | md=30, q_1_=20, q_3_=50, N=139 | 1.00 (0.99-1.01) | .843 |
| Number of days between call 1 and 5 | md=42, q_1_=28, q_3_=67, N=104 | 1.00 (0.99-1.01) | .643 |

^*^ n=number in category, N=total number in analysis.
**^†^** Three questions: 1. The counsellor was understanding and sensitive, 2. The counsellor tried to understand my needs, 3. The counsellor showed respect for my own targets and decisions. Four response alternatives: much, rather, to some extent, not at all. Much for all the three questions was required for “much” in the analysis.
**^‡^** Max number of weeks for any preparation.
^§^ None of these four subjects achieved continuous abstinence
